# A Frog Peptide Ameliorates Skin Photoaging Through Scavenging Reactive Oxygen Species

**DOI:** 10.3389/fphar.2021.761011

**Published:** 2022-01-19

**Authors:** Guizhu Feng, Lin Wei, Helong Che, Yan Shen, Jun Yang, Kai Mi, Jin Liu, Jing Wu, Hailong Yang, Lixian Mu

**Affiliations:** ^1^ School of Basic Medical Sciences, Kunming Medical University, Kunming, China; ^2^ Jiangsu Key Laboratory of Infection and Immunity, Institutes of Biology and Medical Sciences, Soochow University, Suzhou, China; ^3^ Department of General Surgery, the 908th Hospital of Chinese PLA Joint Logistic Support Force, Nanchang, China

**Keywords:** frog, antioxidant peptide, skin photoaging, anti-inflammation, *Nanorana ventripunctata*

## Abstract

Although many bioactive peptides have been identified from the frog skins, their protective effects and the molecular mechanisms against skin photodamage are still poorly understood. In this study, a novel 20-residue peptide (antioxidin-NV, GWANTLKNVAGGLCKMTGAA) was characterized from the skin of plateau frog *Nanorana ventripunctata*. Antioxidin-NV obviously decreased skin erythema, thickness and wrinkle formation induced by Ultraviolet (UV) B exposure in hairless mice. In UVB-irradiated keratinocytes (HaCaT cells) and hairless mice, it effectively inhibited DNA damage through reducing p-Histone H2A.X (γH2AX) expression, alleviated cell apoptosis by decreasing the expression of apoptosis-specific protein (cleaved caspase 3), and reduced interleukin-6 (IL-6) production *via* blocking UVB-activated Toll-like receptor 4 (TLR4)/p38/JNK/NF-κB signaling. In UVB-irradiated human skin fibroblasts (HSF cells) and hairless mice, it effectively restored HSF cells survival rate, and rescued α-SMA accumulation and collagen (especially type I collagen) production by restoring transforming growth factor-β1 (TGF-β1)/Smad2 signaling. We found that antioxidin-NV directly and rapidly scavenged intracellular and mitochondrial ROS in HaCaT cells upon UVB irradiation, and quickly eliminated the artificial free radicals, 2, 2′-azinobis (3-ethylbenzothiazoline-6-sulfonic acid) (ABTS^+^). Taken together, antioxidin-NV directly and rapidly scavenged excessive ROS upon UVB irradiation, subsequently alleviated UVB-induced DNA damage, cell apoptosis, and inflammatory response, thus protecting against UVB-induced skin photoaging. These properties makes antioxidin-NV an excellent candidate for the development of novel anti-photoaging agent.

## Introduction

As the outmost layer of the body, skin is subjected to biotic and abiotic insults such as microorganism infection and radiation injury. Skin tissues can sense environmental regulation of local and overall internal environmental homeostasis through the cutaneous neuro-endocrine system (Slominski et al., 2012). Some stress factors have been shown to affect different cell signaling and biochemical pathways in the skin, for example, ultraviolet (UV) not only triggers mechanisms that protect the integrity of the skin and regulate the overall internal environmental balance, but also triggers skin pathology (aging, cancer, autoimmune reactions) (Slominski et al., 2018). UV radiation causes excessive reactive oxygen species (ROS) formation from UV absorption by non-DNA chromophores in cells (Portugal et al., 2007; Rinnerthaler et al., 2015; Baek and Lee, 2016). The highly reactive molecules are able to damage virtually all categories of cellular constituents including proteins, carbohydrate, lipids, and DNA (Baek and Lee, 2016). The overproduction and/or mismanagement of ROS may result in oxidative stress, which have been implicated in a large variety of skin disorders and skin diseases, such as UV irradiation damages, skin inflammation, bacterial skin infections, and skin cancer (Portugal et al., 2007; Pham-Huy et al., 2008; Godic et al., 2014).

Skin possesses efficient defense mechanisms against oxidative stress under normal conditions, mainly based on the antioxidants. There are two known groups of antioxidant agents, antioxidant enzymes and non-enzymatic low molecular weight antioxidants (LMWAs) (Portugal et al., 2007; Rinnerthaler et al., 2015). The first group is composed of gene-encoded proteins such as superoxide dismutase (SOD), catalase, glutathione peroxidase. The second group is composed of organic small molecules such as glutathione (GSH), carotene, polyphenols, uric acid, CoQ10, vitamin C, vitamin E. No gene-encoded LMWA has been reported until we characterized antioxidant peptides (AOPs) with various structures from the skin secretions of two frog species, *Rana pleuraden* (Yang et al., 2009) and *Odorrana livida* (Liu et al., 2010). Since then, the frog-skin AOPs have been identified by other researchers from different species (Lu et al., 2010; Yu et al., 2015; Barbosa et al., 2018; Niu et al., 2018; Cao et al., 2019; Demori et al., 2019). These data confirm that amphibian skins have a common peptide antioxidant system to cope with the increasing oxidative stress. These frog-skin-derived AOPs are different from antioxidant enzymes and LMWAs. They possess gene-encoded origins as antioxidant enzymes do, but they show no enzyme activity. Instead they function as direct free radical scavengers like LMWAs. The AOPs can rapidly and constantly eliminate the free radicals of ABTS^+^ and/or DPPH that generated by the commercial radical initiators *in vitro* (Yang et al., 2009; Liu et al., 2010; Lu et al., 2010; Yu et al., 2015; Niu et al., 2018; Cao et al., 2019). There is limited understanding of their protective functions and the mechanisms of action against skin injuries caused by ROS *in vivo*. Currently, only two frog-skin AOPs with potential skin protective effects *in vivo* have been described (Qin et al., 2018; Yin et al., 2019). One is antioxidin-RL, which was identified from the frog *Odorrana livida* (Yang et al., 2009); the other is OA-VI12, which was isolated from *O. andersonii* (Cao et al., 2019). They prevented UVB irradiation-induced photoaging in mice, but the detailed mechanisms of the two AOPs remain to be fully understood.

The frogs have developed an excellent chemical defense system composed of various defensive peptides to maintain skin integrity and functionality (Xu and Lai, 2015; Demori et al., 2019). In our previous work, we have characterized a wound healing-promoting peptide, cathelicidin-NV, from the frog skin of *N. ventripunctata*. The peptide effectively accelerated cutaneous wound healing in mice with mechanical injury (Wu et al., 2018). *N. ventripunctata* lives in high altitude (3120–4100 m) where there is low temperature, long sunshine duration and strong ultraviolet radiation. Their naked skins are susceptible to external insults in the harsh environments, especially UV radiation. Based on the wavelength, UV can be classified into three types: UVA (320–400 nm), UVB (280–320 nm), and UVC (100–280 nm). UV irradiation, especially UVB, has the twofold effect of regulating the brain and central neuroendocrine system to rebalance the internal environment (Slominski et al., 2018), and triggering the overproduction of ROS, leading to photo-induced skin damage, skin diseases and even skin cancer (Rinnerthaler et al., 2015). To cope with the increasing oxidative stress, *N. ventripunctata* should possess potent free radical scavenging and radio-protective effect for their survival. Therefore, it is rational to hypothesize that *N. ventripunctata* may also have antioxidant peptide(s) in their skins to protect from the free radicals injury. In this work, we are interested to characterize the peptide antioxidant system from *N. ventripunctata*. Additionally, we try to investigate the potential mechanisms underlying the protective effects of AOP against UVB-induced skin photodamage in hairless mice.

## Materials and Methods

### *N. ventripunctata* Sample

Skin secretions of *N. ventripunctata* (*n* = 30; weight range 20–25 g) were collected as previously reported (Wu et al., 2018). Frogs were stimulated with volatilized anhydrous ether immersed in absorbent cotton, and their skin surface was seen to exude secretions. Skin secretions were washed with 0.1 M phosphate buffer (PBS), (pH 6.0, containing 1% protease inhibitor cocktail, Sigma, United States). The collected solutions containing skin secretions were quickly centrifuged (10, 000 × *g* for 10 min) and the supernatants were lyophilized.

### Peptide Purification

The peptide purification procedures were performed according to the method described in our previous work (Wu et al., 2018). An aliquot (1 g) of lyophilized skin secretion was dissolved in 10 ml PBS and centrifuged at 5, 000 × *g* for 10 min. The supernatant was applied to a Sephadex G-50 (Superfine, Amersham Biosciences) gel filtration column (2.6 cm diameter, 100 cm length) equilibrated with 0.1 M PBS for preliminary separation. Elution was performed with the same buffer, collecting fractions of 3.0 ml. The absorbance of the eluted fractions were monitored at 280 nm. The anti-photoaging activity in mice was tested as described below. The fraction containing anti-photoaging activity was further purified by a C_18_ reversed-phase high performance liquid chromatography (RP-HPLC, Gemini C_18_ column, 5 μm particle size, 110 Å pore size, 250 mm length, 4.6 mm diameter) column. The elution is performed using a linear gradient of 0–80% acetonitrile containing 0.1% (v/v) trifluoroacetic acid in 0.1% (v/v) trifluoroacetic acid/water over 60 min as illustrated in Supplementary Figure S1B. UV-absorbing peaks were collected, lyophilized, and assayed for anti-photoaging activity. Peaks with anti-photoaging activity were collected and lyophilized for a second HPLC purification procedure using the same condition as illustrated in Supplementary Figure S1C.

### Primary Structural Analysis

N-terminal sequence of the purified peptide was determined by Edman degradation on an Applied Biosystems pulsed liquid-phase sequencer (model ABI 491). Matrix-assisted laser desorption/ionization time-of-flight mass spectrometry (MALDI-TOF MS) was used to identify the purity of the isolated peptide. AXIMA CFR mass spectrometer (Kratos Analytical) was analyzed in linear and positive ion mode using an acceleration voltage of 20 kV and an accumulating time of single scanning of 50 s.

### cDNA Cloning

The experiment was performed according to the method described in our previous work (Wu et al., 2018). Total RNA was extracted from the skin of *N. ventripunctata* using RNeasy Protect Mini Kit (QIAGEN, Germany) according to the manufacturer’s instructions. An in-fusion SMARTer™ directional cDNA library construction kit was used for cDNA synthesis. The synthesized cDNA was used as template for PCR to screen the cDNAs encoding the purified peptide (antioxidin-NV). According to the sequence determined by Edman degradation, an antisense degenerate primer (antioxidin-NV-R1) was designed and coupled with a 5′ PCR primer (the adaptor sequence of 3′ PCR primer provided in the kit) to screen the 5′ fragment of cDNA encoding antioxidin-NV. Then, a sense primer (antioxidin-NV-F1) was designed according to the 5′ fragment and coupled with 3′ PCR primer from the kit to screen the full-length cDNAs. The PCR conditions were, 2 min at 95°C, and 30 cycles of 10 s at 92°C, 30 s at 50°C, 40 s at 72°C followed by 10 min extension at 72°C. The PCR products were cloned into pGEM^®^-T easy vector (Promega, Madison, WI, United States). DNA sequencing was performed on an Applied Biosystems DNA sequencer, model ABI PRISM 377. Primers used in this research are listed in the supplementary material Supplementary Table S1.

### Peptide Synthesis

Antioxidant-NV (GWANTLKNVAGGLCKMTGAA) and the scrambled version of antioxidin-NV called sNV (LTAGMAWNAKGKACTVGLGN), were synthesized by the peptide synthesizer Synpeptide Co. Ltd (Shanghai, China). The synthetic peptides were purified and then analyzed by HPLC and MALDI-TOF MS to confirm that the purity was higher than 98%.

### ABTS^+^ Scavenging

Free radical scavenging activity was determined by measuring reduction of radical 2, 2′-azinobis (3-ethylbenzothiazoline-6-sulfonic acid) (ABTS^+^) according to manufacture instruction of the kit GMS10114.4 (Genmed Scientifics INC, Shanghai, China). The total formation of products (*i.e*. the reduced form of ABTS and the purple antioxidin-NV modification) and the total consumption of ABTS radical were determined by linear regression analysis. The concentrations of ABTS and ABTS free radical were calculated by using ε_340_ = 4.8 × 10^4^ M^−1^cm^−1^ and ε_415_ = 3.6 × 10^4^ M^−1^cm^−1^, respectively (Yu et al., 2015). The purple antioxidin-NV modification was monitored at A_550_.

### Cytotoxicity and Hemolysis

Cytotoxicity against human skin fibroblasts (HSFs) (KCB 200537, Kunming Cell Bank, Chinese Academy of Sciences) and human HaCaT keratinocytes (KCB200442YJ, Kunming Cell Bank, Chinese Academy of Sciences) was determined by the MTT assay. Antioxidin-NV dissolved in serum-free DMEM medium was added to cells in 96-well plates (2 × 10^4^ cells/well), and the serum-free DMEM medium without antioxidin-NV was used as control. After incubation for 24 h, 20 μl of MTT solution (5 mg/ml) was added to each well, and the cells were further incubated for 4 h. Finally, cells were dissolved in 200 μl of Me_2_SO_4_, and the absorbance at 570 nm was measured. Rabbit erythrocyte suspensions were incubated with antioxidin-NV and then the absorbance of supernatant was measured at 540 nm. 1% (v/v) Triton X-100 and PBS were used as positive and negative controls, respectively (Mu et al., 2017).

### Determination of Intracellular and Mitochondrial ROS Production

The level of intracellular ROS generation was detected using 2′, 7′-dichlorodihydrofluorescein diacetate (DCFH-DA) with an Ex/Em of 504/529 nm. After 24 h later with UVB irradiation and sample treatment, cells were stained with 30 μM 2′, 7′- DCFH-DA (Sigma, United States) for 30 min at 37°C in a CO_2_ incubator. The cells were then analyzed by flow cytometry (FACSCaliburTM, Becton-Dickinson, CA, United States) and an inverted fluorescence microscope (Zeiss, Germany). Mitochondrial ROS production with an Ex/Em of 585/590 nm was detected using mitochondrial reactive oxygen ROS kit (CA1310, Solarbio, China) as described by the manufacturer’s instructions.

### UVB Irradiation and Antioxidin-NV Treatment in Cells

UVB irradiation and sample treatment were performed according to a method previously reported (Hwang et al., 2013a; Hwang et al., 2013b). When HaCaT or HSF cells were cultured in six-well culture plates (2 × 10^6^ cells/well) and reached over 80% coverage, cells were pretreated with serum-free DMEM for 12-h incubation, then cells were washed twice with phosphate buffered saline (PBS). The cells with thin layers of PBS were exposed to UVB lamps (JT8-Y20W, Philips, Netherlands) in the wavelength range of 280–320 nm and their irradiation intensity was measured with a UVB irradiometer (Shanghai Sigma High Technology Co. Ltd, Shanghai, China), controlling the total irradiation dose at 80 mJ/cm^2^. After UVB irradiation, the cells were washed with warm PBS three times. The cells were immediately treated with antioxidin-NV (10, 20, and 40 μg/ml) or vitamin C (40 μg/ml, SCR, China) in serum-free medium conditions for 24 h. Control cells were maintained in the same culture conditions without UVB exposure.

### DNA Fragmentation Analysis

DNA fragmentation was assayed by agarose gel electrophoresis. HaCaT cells were seeded in six-well plates and cultured as described above. After 24 h later with UVB irradiation and sample treatment, HaCaT cells DNA were extracted for DNA fragmentation analysis. Cellular DNA was extracted using cell genomic DNA extraction kit (Solarbio, China) as described by the manufacturer’s instructions. The DNA samples were mixed with the 6× loading buffer (TaKaRa, Japan) and stained with nucleic acid dye (ZEESAN, China), and then used 10 μl for 1% agarose gel electrophoresis and observed under UV light imaging system (Bio-Rad ChemiDoc™ XRS, United States).

### Western Blot Analysis

After 24 h later with UVB irradiation and sample treatment, the cells were washed twice with ice-cold PBS and lysed with RIPA lysis buffer (Beyotime, China). The proteins were extracted for western blot analysis according to our previously described method (Wu et al., 2018). The concentration of protein was determined by the Bradford protein assay. Then the cellular proteins were separated on a 12% SDS-PAGE gel and electro blotted onto a polyvinylidene difluoride membrane. Primary antibodies against γH2AX, JNK, p38 MAPK, IkBα, NF-κB p65, caspase-3, cleaved caspase-3, Smad2 (1:2000; CST, United States), and β-actin (1:5000, Santa Cruz Biotechnology, United States) were used in western blot analysis.

### Immunofluorescence Staining

HaCaT cells were seeded in 24-well plates (5 × 10^5^ cells/well) with cell crawling (Solarbio, China) and cultured as described above. After 24 h later with UVB irradiation and sample treatment, the cells were washed twice with ice-cold PBS, then treated with 0.5% Triton X-100 (Solarbio, China) for 15 min, and then blocked with 5% BSA (Solarbio, China) for 2 h, followed by an overnight incubation with a primary antibody against cleaved caspase 3 antibody (1:400, CST, United States), Phospho-Histone H2A.X (1:400, CST, United States) at 4°C, respectively. The experiments were conducted with anti-rabbit IgG-FITC (1:100, Solarbio, China) for 1 h at room temperature using DAPI-containing mounting tablets (Solarbio, China). The pictures were collected using an inverted fluorescence microscope (Zeiss, Germany).

Skin tissues in UVB-irradiated hairless mice were taken for tissue immunofluorescence staining. Primary antibodies against cleaved caspase 3, collage I (1:400, CST, United States) were used in tissue immunofluorescence analysis.

### Apoptosis in Flow Cytometry

Annexin V-fluorescein isothiocyanate (FITC)/propidium iodide (PI) double staining was used to measure percentile of apoptosis in HaCaT cells. After 24 h later with UVB irradiation and sample treatment, the cells were re-suspended in 500 μl of 1× binding buffer and mixed with Annexin V-FITC/PI (Cat number APOAF, Sigma, United States). After incubation for 30 min, the cells were measured by Accuri C6 flow cytometry (Accuri, Ann Arbor, United States).

### Cytokine and Chemokine Measurements

After 24 h later with UVB irradiation and sample treatment, culture supernatants were collected and assessed for transforming growth factor-β1 (TGF-β1) and IL-6 using ELISA kits (DAKAWE, Beijing, China).

Photo-aged skin tissue weighing 100 mg plus ice-cold PBS was fully ground into a 10% (m/v) tissue suspension. The suspension was processed by an ultrasonic disruptor (Saifei, China), centrifuged at 4°C for 10 min (3500 g/min), and the supernatant was collected. The supernatant was used to assay the level of TGF-β1 and IL-6 using ELISA kits (DAKAWE, Beijing, China).

### Experimental Animals and Ethics Statement

Adult *N. ventripunctata* (*n* = 30; weight range 20–25 g) was collected from Shangri-La, Yunnan province of China. Adult male SKH-1 hairless mice were purchased from Labreal Laboratories and housed in the pathogen-free facility. At the termination of the study, mice were sacrificed by cervical dislocation under CO_2_ anesthesia in accordance with the guidelines from the Care and Use of Medical Laboratory Animals (Ministry of Health, People’s Republic of China). All the animal study was reviewed and approved by the Institutional Animal Care and Use Ethics Committee of Kunming Medical University (IACUC approval number: KMMU2020063). All the animal experiments described in this study were conducted at Kunming Medical University.

### Hairless Mouse Model of Photoaged Skin and Antioxidin-NV Treatment

Adult male SKH-1 hairless mice (*n* = 30, 6–8 weeks old, 20–30 g, Labreal Laboratories) were used. The mice were housed for at least 7 days prior to the experiments in a ventilated and temperature-controlled room and had access to water ad libitum. ASS-03AB UV phototherapy light source (Shanghai Sigma High Technology Co. Ltd, Shanghai, China) was used for UVB irradiation (wavelength 280–320 nm). The mice were randomized into five treatment groups (six mice per group): (Slominski et al., 2012) Sham (mice were covered with PBS); (Slominski et al., 2018) PBS (mice were covered with PBS after UVB exposure); (Portugal et al., 2007) NV (mice were covered with antioxidin-NV dissolved in PBS after UVB exposure); (Rinnerthaler et al., 2015) VC (mice were covered with vitamin C dissolved in PBS after UVB exposure, vitamin C, recognized as an antioxidant, is often used to prevent light-induced skin aging, therefore, vitamin C was selected as the positive control) and (Baek and Lee, 2016) sNV(the scrambled version of antioxidin-NV, mice were covered with sNV dissolved in PBS after UVB exposure). In the PBS, NV, VC and sNV group, mice were directly exposed to UVB radiation, then were treated with PBS, antioxidin-NV, vitamin C, or sNV (100 μl, 200 μg/ml) to the back, respectively. Mice were exposed to UVB radiation at 100 mJ/cm^2^ (one minimal erythematal dose = 100 mJ/cm^2^) five times during the first week and then to 200 mJ/cm^2^ three times a week for 12 weeks thereafter. After sacrifice, some of the skin tissues were snap frozen in liquid nitrogen and stored at −80°C, and others were fixed in formalin and embedded in paraffin for immunohistochemistry.

### Histological Analysis

The tissues were fixed in 10% formalin. Then, the tissues were sectioned using a microtome and stained with hematoxylin and eosin (H&E) for histological analysis. The pathology slides were read in blindness, and the images were recorded.

### Masson Stain

The paraffin-embedded skin specimens were measured using Masson’s trichrome stain kit (Solarbio, China). The slides were stained with Bouin’s Fluid and Weigert’s iron hematoxylin working solution. Furthermore, the slides were differentiated in phosphomolybdic-phosphotungstic acid solution and stained with aniline blue solution. Finally, the slides were read in blindness, and the images were recorded.

### Immunohistochemistry (IHC) Analysis

The paraffin-embedded tissue sections were dried, deparaffinized, and rehydrated. Following a microwave pretreatment in citrate buffer (pH 6.0), the slides were immersed in 3% hydrogen peroxide for 20 min to block the activity of endogenous peroxidase. After extensive washing with PBS, the slides were incubated with γH2AX (1:480; CST, United States), Cleaved Caspase 3 antibody (1:200; CST, United States), Collagen I antibody (1:200; abcam, United Kingdom) or α-SMA antibody (1:100; abcam, United Kingdom) overnight at 4°C. The sections were then incubated with the secondary antibody for 1 h at room temperature, and the slides were developed using the UltraVision Quanto HRP detection kit (Thermo Scientific, United States). Finally, the slides were counterstained using hematoxylin. The slides were read in blindness, and the images were recorded.

### Statistical Analysis

Statistical differences were determined using Student’s *t*-tests or one-way ANOVA provided by GraphPad Prism software. Results are shown as mean ± SD from three independent experiments. A *p* value less than 0.05 was considered as statistically significant difference.

## Results

### Isolation and Characterization of Antioxidin-NV

As shown in Supplementary Figure S1A, the skin secretions of *N. ventripunctata* were divided into five fractions after Sephadex G-50 gel filtration. The fraction containing anti-photoaging activity was pooled and subjected to a C_18_ RP-HPLC column for further purification (Supplementary Figures S1B,C). The purified peptide was designated as antioxidin-NV (Supplementary Figure S1C). After Edman degradation, the amino acid sequence of antioxidin-NV was identified as GWANTLKNVAGGLCKMTGAA. MALDI-TOF MS analysis indicated that antioxidin-NV had a measured molecular mass of 1963.70 Da (Supplementary Figure S2), matching well with the calculated molecular mass of 1963.30 Da.

**FIGURE 1:**
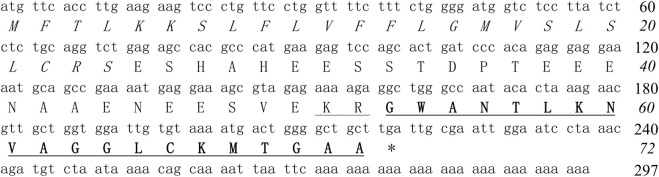
The cDNA sequence of antioxidin-NV precursor. Deduced amino acid sequence is shown below the cDNA sequence. The putative signal peptide is italicized and the amino acid sequence of mature peptide is underlined and bold. The stop codon is indicated by an *asterisk*. Amino acid numbers or nucleotide numbers are shown after the sequences.

The cDNA clone encoding the precursor of antioxidin-NV was sequenced from the skin cDNA library of *N. ventripunctata* (GenBank accession number: MW114946). As shown in Figure 1, the deduced amino acid sequence of antioxidin-NV is completely consistent with that sequenced by Edman degradation. It is composed of 72 amino acid residues, including a predicted signal peptide (24 amino acid residue), an acidic peptide region (28 amino acid residue) that ends in a typical trypsin-like proteases processing site (-Lys^51^Arg^52^-), followed by a mature peptide (20 amino acid).

### Antioxidin-NV Rapidly Eliminated Artificial ABTS^+^ Radicals and Scavenged Intracellular/Mitochondrial ROS

**FIGURE 2:**
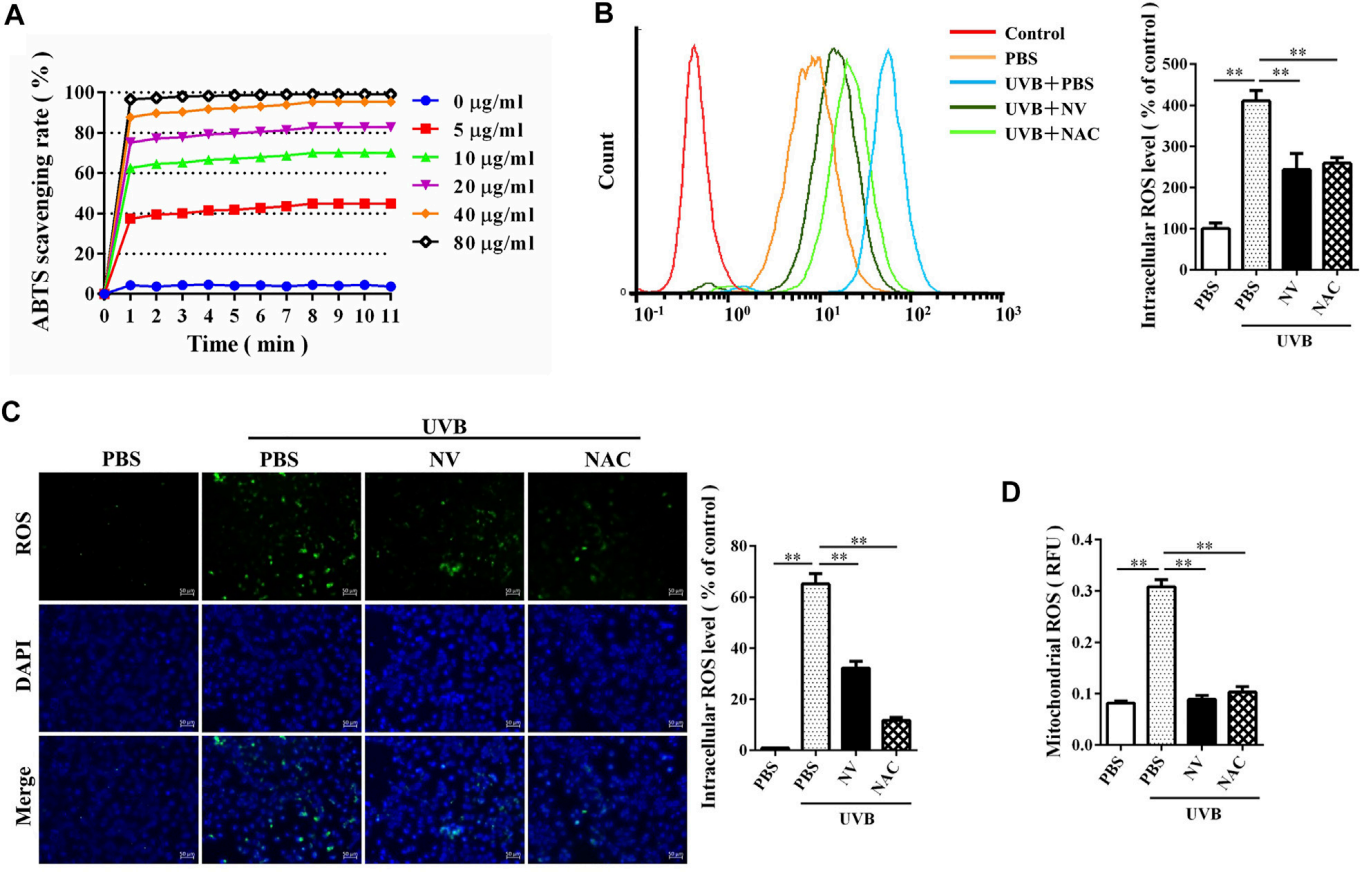
Antioxidin-NV rapidly scavenged UVB-induced intracellular and mitochondrial ROS for HaCaT cells, and eliminated artificial free radicals ABTS^+^. **(A)** Percent rate of ABTS^+^ was scavenged by antioxidin-NV in a dose and time dependant manner. Antioxidin-NV showed strong antioxidant activities. **(B)** Antioxidin-NV suppressed intracellular ROS generation. ROS generation levels were determined by treating the samples for 24 h after UVB irradiation. DCFH-DA (10 μM) was introduced into HaCaT cells, and fluorescence was measured by flow cytometry. **(C)** DCFH-DA fluorescence in HaCaT cells was measured by confocal microscopy. Intracellular ROS is stained green. Scale bar = 100 μm. **(D)** Antioxidin-NV suppressed mitochondria ROS generation for HaCaT cells. NAC (ROS inhibitor) was used as a positive control; Data are presented as mean ± SD (*n* = 3). ns, no significance, ^**^ *p* < 0.01, ^***^ *p* < 0.001.

ABTS^+^ free radical scavenging kinetics, owing to its relative stability, easy measurement, good reproducibility, ABTS^+^ radicals are commonly used to evaluate antioxidant capacity (Yang et al., 2009). We confirmed the antioxidant activity of antioxidin-NV by assessing its ability to scavenge ABTS^+^ free radical. The assay is based on decolorization by monitoring absorbance decreases at the characteristic wavelength of 734 nm. As illustrated in Figure 2A, antioxidin-NV could rapidly scavenge ABTS^+^ in a dose-dependent manner. It could get rid of ABTS^+^ immediately when it contacted with ABTS^+^. At the concentration of 80 μg/ml, antioxidin-NV scavenged 96% ABTS^+^ within 1 min, and scavenged nearly 99% ABTS^+^ within 8 min. Even the concentration down to 5 μg/ml, 40% ABTS^+^ was scavenged within 4 min by antioxidin-NV.

Then, we were interested to assay whether antioxidin-NV directly clear the ROS induced by UVB irradiation in HaCaT cells. As an indicator of ROS production, DCFH-DA fluorescence intensity was measured by flow cytometry. A progressive increment of intracellular ROS level was observed in the UVB-irradiated HaCaT cells, and the addition of antioxidin-NV significantly decreased the intracellular ROS level in HaCaT cells upon UVB irradiation (Figures 2B,C). The scavenging efficacy of UVB-induced intracellular ROS is comparable to the ROS inhibitor, N-acetyl-L-cysteine (NAC) (Figures 2B,C). Furthermore, antioxidin-NV effectively cleared the ROS in mitochondria induced by UVB irradiation (Figure 2D). The data indicate that antioxidin-NV had a strong ability to scavenge ROS induced by UVB irradiation, suggesting a strong antioxidant activity of antioxidin-NV.

### Antioxidin-NV Suppressed UVB-Induced Skin Photoaging in Hairless Mice

**FIGURE 3:**
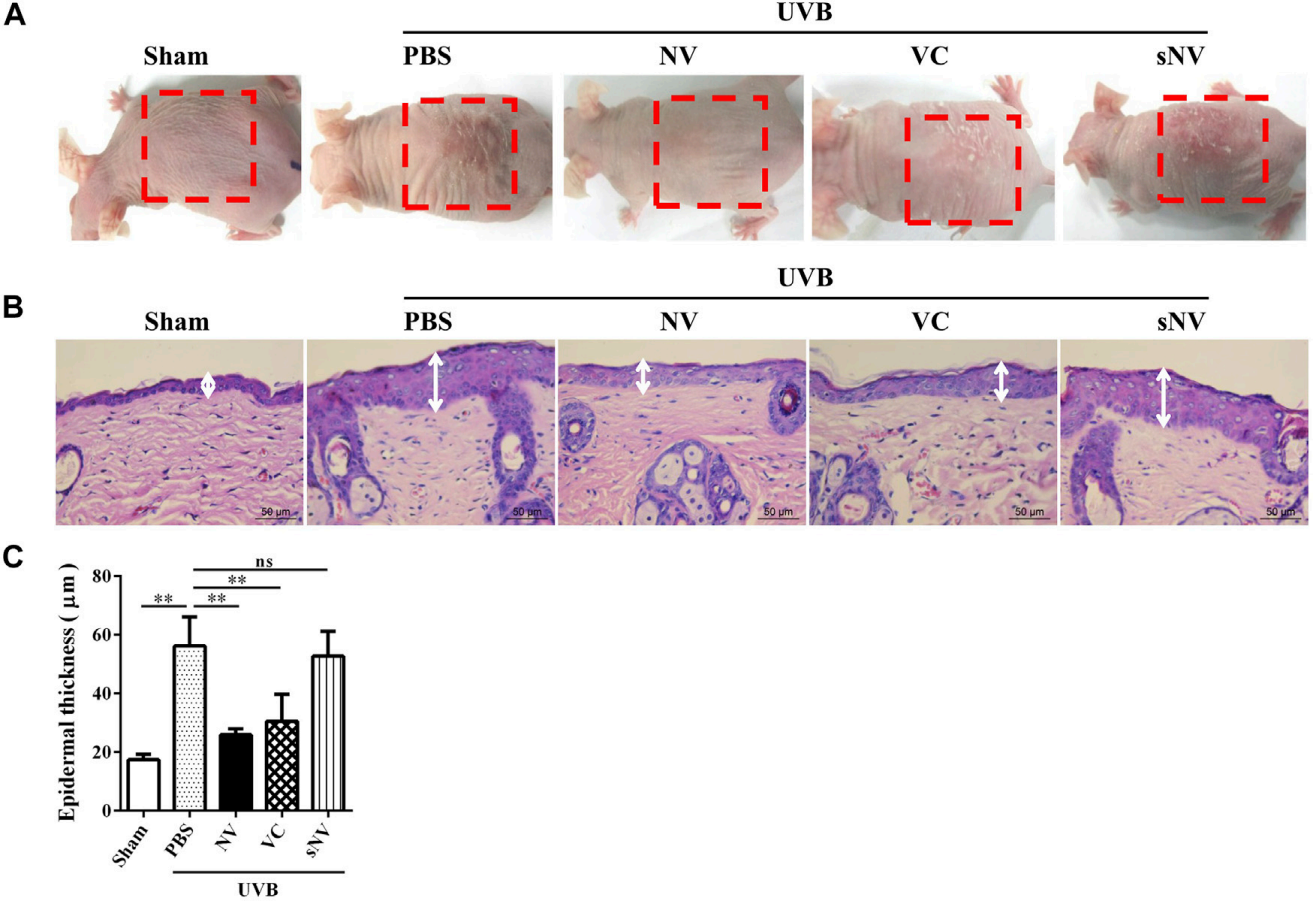
Topical application of antioxidin-NV significantly suppressed UVB-induced skin photoaging in hairless mice, obviously decreased skin erythema, thickness and wrinkle formation. **(A)** Images of a representative mouse from each group taken after 12 weeks are shown. 200 mJ/cm^2^ UVB radiation and vehicle, antioxidin-NV, VC or sNV (the scrambled version of antioxidin-NV) were used to treat the back skin of mice for 12 weeks. The red dotted line indicated the UVB-induced skin photoaging: decreased skin erythema, coarse wrinkling, rough texture and thickening. **(B)** skin tissues were taken and paraffin blocks were cut into 4 μm thick sections for HE staining, and the white line indicated the thickness of the epidermis. Scale bar = 50 μm. **(C)** Epidermal thicknesses in each group of mice were measured and analyzed. Data are presented as mean ± SD (*n* = 6). ns, no significance, ^**^ *p* < 0.01.

UV-induced skin photoaging leads to the accumulation of intracellular ROS (Zhang et al., 2017), especially the stronger biological effect of UVB (Diffey, 2002). To evaluate the anti-phtoaging activity of antioxidin-NV, we established a UVB-induced skin photoaging mouse model to assay whether topical application of antioxidin-NV can inhibit skin photoaging in mice. As illustrated in Figure 3A, UVB irradiation obviously induced skin photoaging in hairless mice, but topical application of antioxidin-NV significantly suppressed UVB-induced skin photoaging in hairless mice with reduced skin erythema, hyperplasia, wrinkling, and roughness compared to PBS-treated mice. H&E staining of the dorsal skin showed that UVB-irradiation resulted in a reduction of the thickness of epidermal layers, but topical application of antioxidin-NV significantly reversed this reduction (Figures 3B,C). To our surprise, antioxidin-NV showed a better therapeutic efficacy against UVB-induced skin photoaging than vitamin C (VC, positive control) (Figures 3A–C). The scrambled antioxidin-NV (sNV, isotype control) had no significant therapeutic effects on UVB-induced skin photoaging, indicating that the therapeutic efficacy of antioxidin-NV against UVB-induced skin photoaging is due to its unique amino acid sequence (Figures 3A–C). Besides, antioxidin-NV did not exhibit cytotoxicity and hemolytic activity at an absolutely high concentration (Supplementary Figure S3), and no adverse effect on the body weight, general health or behavior of the mice were observed for the topical antioxidin-NV treatment, implying antioxidin-NV had low side effects.

### Antioxidin-NV Inhibited UVB-Induced DNA Damage in HaCaT Cells and Hairless Mice by Reducing p-Histone H2A.X (γH2AX) Expression

**FIGURE 4:**
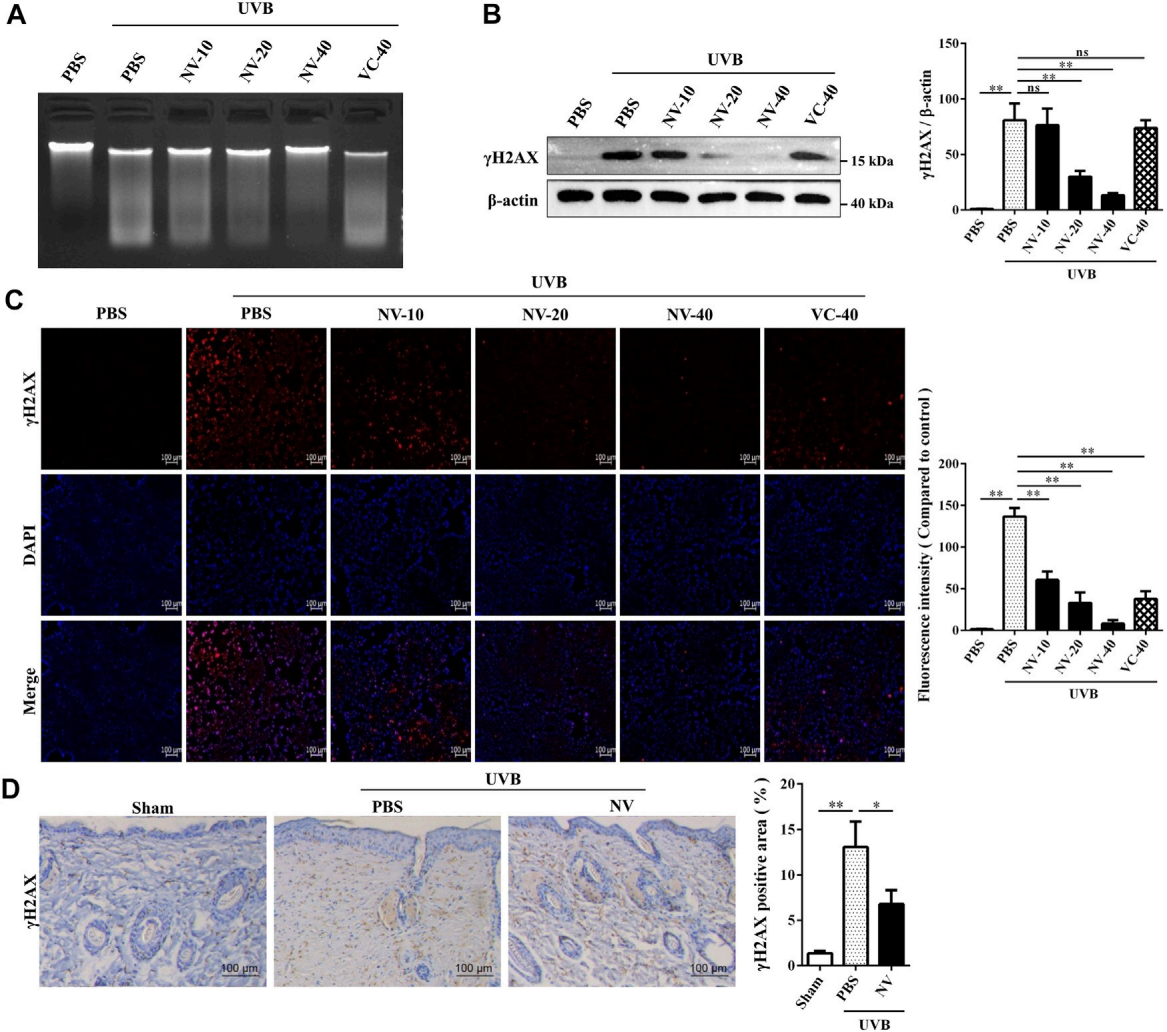
Antioxidin-NV inhibited UVB-induced DNA damage *in vivo* and *in vitro*. **(A)** HaCaT cells were treated with samples for 24 h after UVB irradiation and HaCaT cells DNA were extracted for DNA fragmentation analysis. **(B)**: Western blot was performed to analyze the expression of γH2AX proteins in HaCaT cells. relative activation analysis were quantified by Image J. **(C)** The expression of γH2AX in HaCaT cells was analyzed by cell immunofluorescence analysis. γH2AX proteins are stained red. Scale bar = 100 μm. **(D)** Immunohistochemical staining of skin tissues was used to determine the expression levels of γH2AX in HaCaT cells. γH2AX proteins are stained brown (Indicated by red arrow). Scale bar = 100 μm. Data are presented as mean ± SD (*n* = 3). NV-10, 20, 40, VC-40 indicated the concentrations of 10, 20, 40 μg/ml respectively. ns, no significance, ^*^ *p* < 0.05, ^**^ *p* < 0.01.

Skin photoaging is closely associated with DNA damage (Zhang et al., 2017), and keratinocytes are the cells distributed in the outer layer of skin which can be directly irradiated by UVB. So we analyzed whether antioxidin-NV can suppress UVB-induced DNA damage. Agarose gel electrophoresis showed that UVB irradiation produced a typical ladder with clearly increased intensity of DNA fragmentation in HaCaT cells, and antioxidin-NV significantly reduced its formation in a dose-dependent manner (Figure 4A). Western blot analysis and immunofluorescence staining further showed that UVB irradiation resulted in a significant increment of p-Histone H2A.X (γH2AX) expression, a marker protein for DNA damage. However, antioxidin-NV significantly reduced γH2AX expression in HaCaT cells induced by UVB irradiation in a dose-dependent manner (Figures 4B,C). Furthermore, IHC analysis also showed that UVB irradiation significantly increased γH2AX expression in hairless mice, but topical application of antioxidin-NV reduced UVB-induced γH2AX expression (Figure 4D).

### Antioxidin-NV Inhibited UVB-Induced Cell Apoptosis in HaCaT Cells and Hairless Mice

**FIGURE 5:**
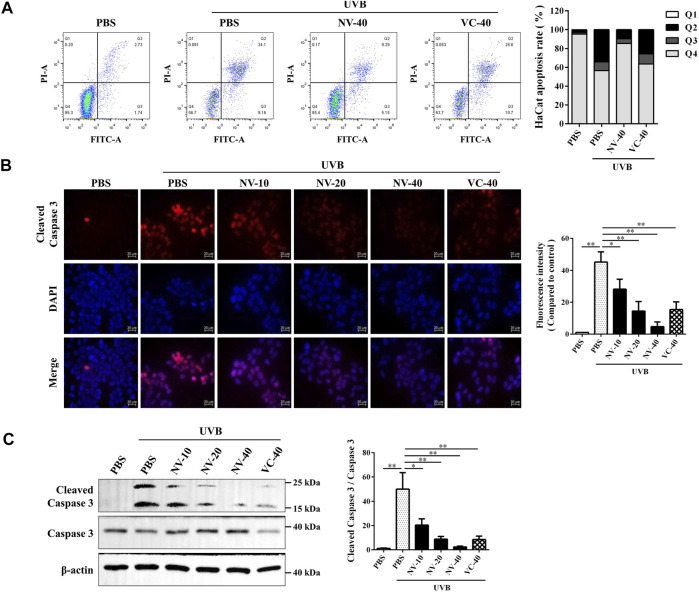
Antioxidin-NV inhibited UVB-induced apoptosis and the expression of apoptotic protein Cleaved Caspase 3 in HaCaT cells. **(A)** HaCaT cells were treated with samples for 24 h after UVB irradiation and HaCaT cell apoptosis was analyzed by flow cytometry. **(B)** The expression of Cleaved Caspase 3 in HaCaT cells was analyzed by cell immunofluorescence analysis. Cleaved Caspase 3 proteins are stained red. Scale bar = 20 μm. **(C)** Western blot was performed to analyze the expression of Caspase 3 and Cleaved Caspase 3 proteins in HaCaT cells. relative activation analysis were quantified by Image J. Data are presented as mean ± SD (*n* = 3). NV-10, 20, 40, VC-40 indicated the concentrations of 10, 20, 40 μg/ml respectively. ^*^ *p* < 0.05, ^**^ *p* < 0.01.

Cell apoptosis is a critical pathological process of skin photoaging (Liu et al., 2021). The therapeutic effect of antioxidin-NV against UVB-induced apoptosis was assayed by flow cytometry. As illustrated in Figure 5A, UVB exposure resulted in the apoptosis of HaCaT cells (43.26%), while antioxidin-NV (40 μg/ml) treatment reduced UVB-induced HaCaT cell apoptosis at both the early and late stages (14.44%). Immunofluorescence and western blot analysis showed that antioxidin-NV significantly reduced the expression of apoptotic protein cleaved caspase 3 (a marker protein for apoptosis) in the UVB-irradiated HaCaT cells in a dose-dependent manner (Figures 5B,C).

**FIGURE 6:**
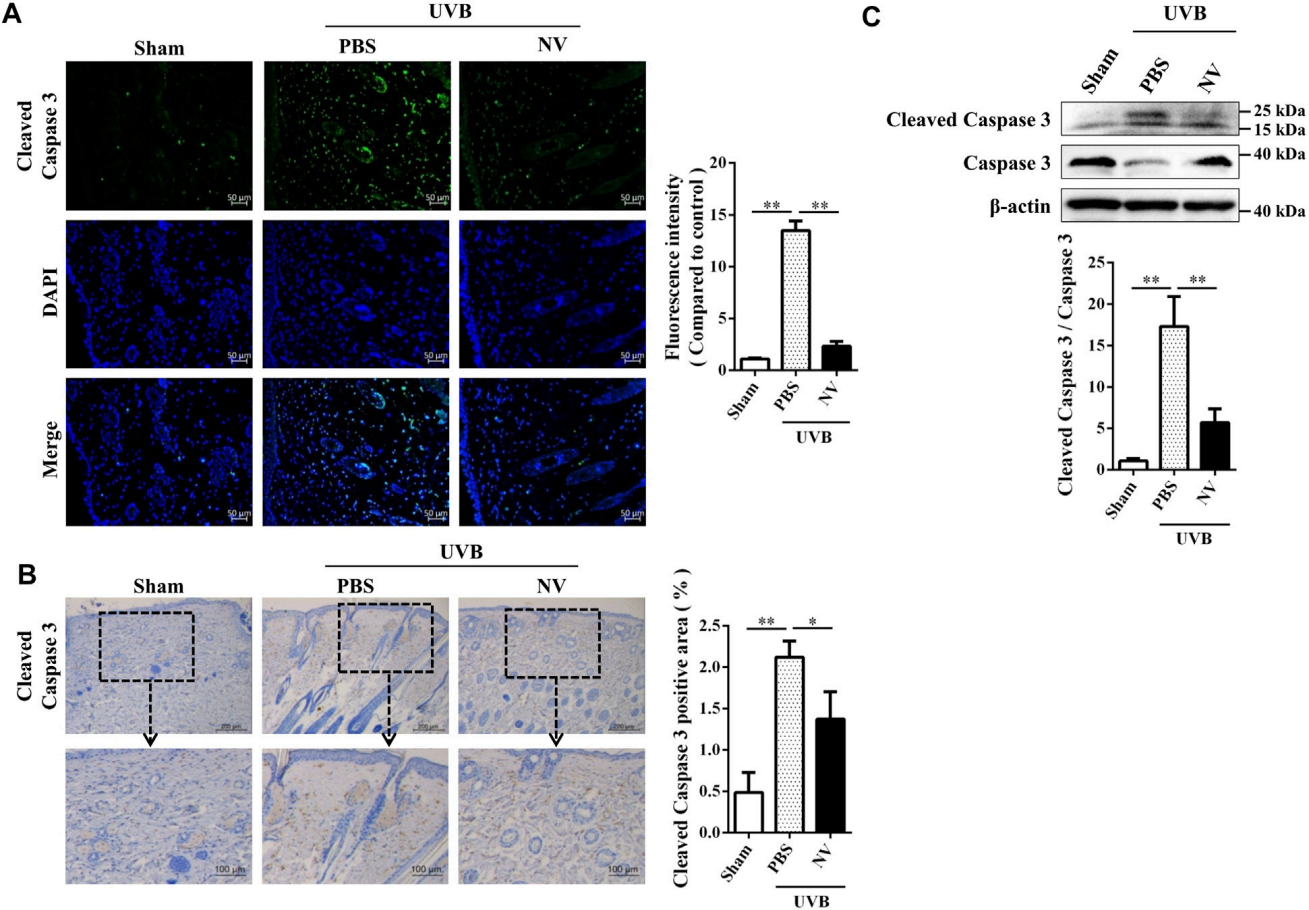
Antioxidin-NV inhibited UVB-induced apoptosis in the skin of hairless mice. **(A)** skin tissues were taken and paraffin blocks were cut into 4 μm thick sections for anti-Cleaved Caspase 3 immunofluorescence. Cleaved Caspase 3 proteins are stained green. Scale bar = 50 μm. **(B)** Immunohistochemical staining of skin tissues was used to determine the expression levels of Cleaved Caspase 3. Cleaved Caspase 3 proteins are stained brown (Indicated by red arrow). Scale bar = 200 μm. **(C)** Western blot was performed to analyze the expression of anti-Caspase 3 and anti-Cleaved Caspase 3 proteins and relative activation analysis was quantified by Image J. Data are presented as mean ± SD (*n* = 3). ^*^ *p* < 0.05, ^**^ *p* < 0.01.

We further analyzed whether antioxidin-NV can suppress UVB-induced cell apoptosis in hairless mice. Immunofluorescence, IHC and western blot analysis showed that UVB irradiation markedly increased the expression of cleaved caspase-3 in the skin of hairless mice, indicating that UVB irradiation significantly resulted in cell apoptosis in the skin of mice (Figures 6A–C). But antioxidin-NV significantly inhibited the expression of cleaved caspase-3 in the skin of hairless mice induced by UVB irradiation, suggesting that it could inhibit UVB-induced cell apoptosis in the skin of hairless mice (Figures 6A–C).

### Antioxidin-NV Inhibited UVB-Induced Inflammatory Response in HaCaT Cells and Hairless Mice by Attenuating UVB-Activated TLR4/p38/JNK/NF-κB Signaling

**FIGURE 7:**
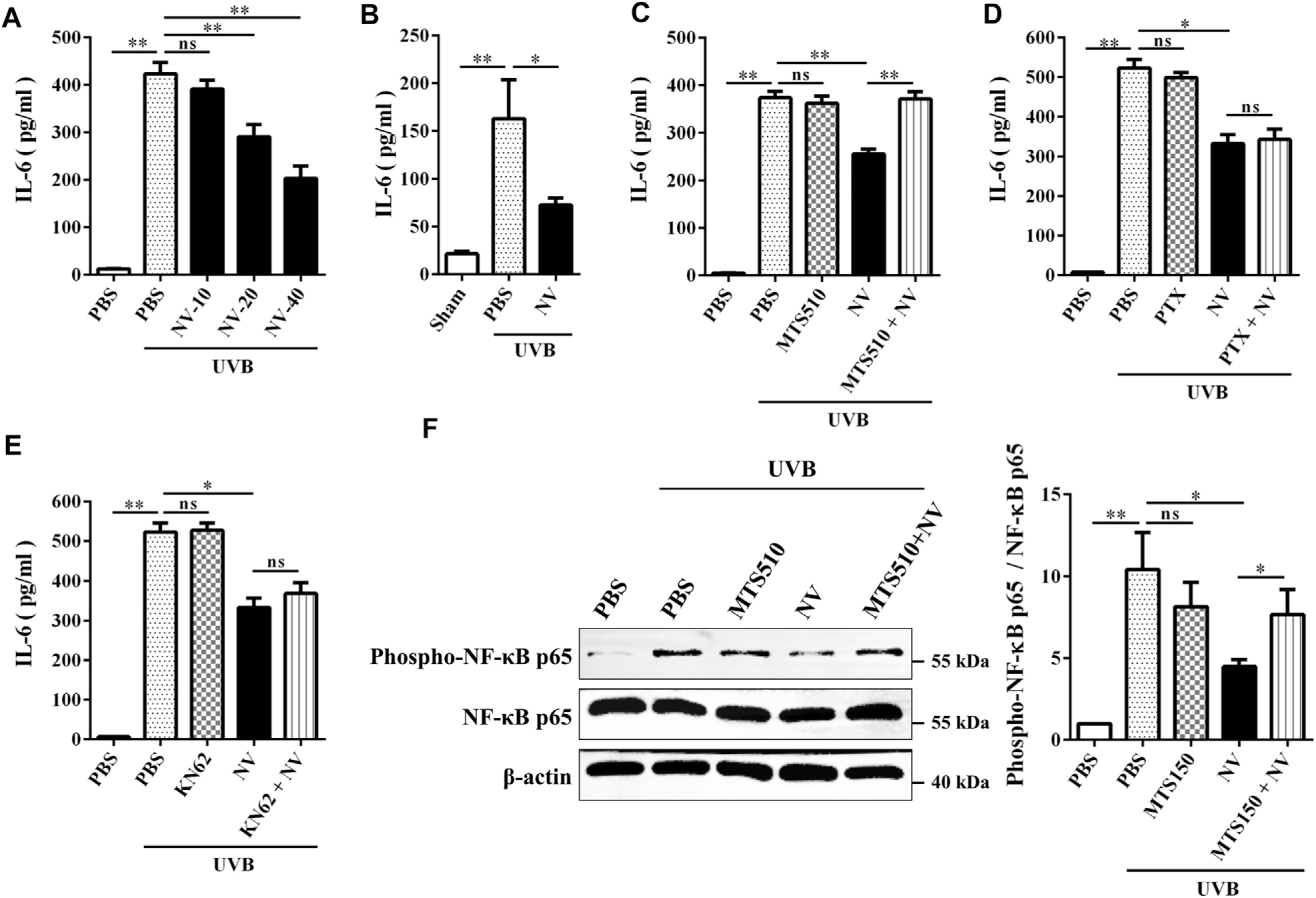
Antioxidin-NV inhibited UVB-induced IL-6 expression by inhibiting toll-like receptor 4 (TLR4). **(A**,**B)** Antioxidin-NV significantly decreased IL-6 secretion in UVB exposed HaCaT cells **(A)** and UVB exposed hairless mice skin **(B)**. **(C**–**E)** Toll-like receptor (TLR) 4 mediate the antioxidin-NV-induced IL-6 down-regulation. MTS510, toll-like receptor (TLR) 4 inhibitor; KN-62, the purine receptor inhibitor; PTX, G protein coupling receptor inhibitor. **(F)** Western blot showed effects of antioxidin-NV on P65 protein kinases phosopholyation in HaCaT cells and relative activation analysis. Data are presented as mean ± SD (*n* = 3). NV-10, 20, 40 indicated the concentrations of 10, 20, 40 μg/ml respectively. ns, no significance, ^*^ *p* < 0.05, ^**^ *p* < 0.01.

Inflammation was found to enhance the epidermal hyperproliferative response to UVB and play a crucial role in promoting skin photoaging (Pillai et al., 2005). As illustrated in Figure 7A, UVB irradiation obviously increased the secretion of IL-6 in UVB-exposed HaCaT cells, but antioxidin-NV effiectively suppressed the secretion of IL-6 in a dose-dependent manner. Furthermore, UVB irradiation increased IL-6 production in the skins of hairless mice, but the topical application of antioxidin-NV significantly decreased the secretion of IL-6 compared to PBS treatment (Figure 7B).

In addition, inflammation-related receptor inhibitors were used to determine which receptor was involved in antioxidin-NV-mediated IL-6 down-regulation in UVB-irradiated HaCaT cells (Figures 7C–E). After the addition of TLR4 inhibitor MTS510 (10 μg/ml), antioxidin-NV-mediated IL-6 down-regulation in UVB-irradiated HaCaT cells was completely inhibited (Figure 7C). The purine receptor inhibitor KN-62 (2 μM) and G protein coupling receptor inhibitor PTX (10 μg/ml) had no significant effect on antioxidin-NV-mediated IL-6 down-regulation (Figures 7D,E). These results suggest that TLR4 is involved in the antioxidin-NV-mediated IL-6 down-regulation in UVB-irradiated HaCaT cells. In addition, after the treatment by TLR4 inhibitor MTS510, the inhibitory effect of antioxidin-NV on NF-κB p65 phosphorylation in UVB-irradiated HaCaT cells was completely inhibited (Figure 7F).

**FIGURE 8:**
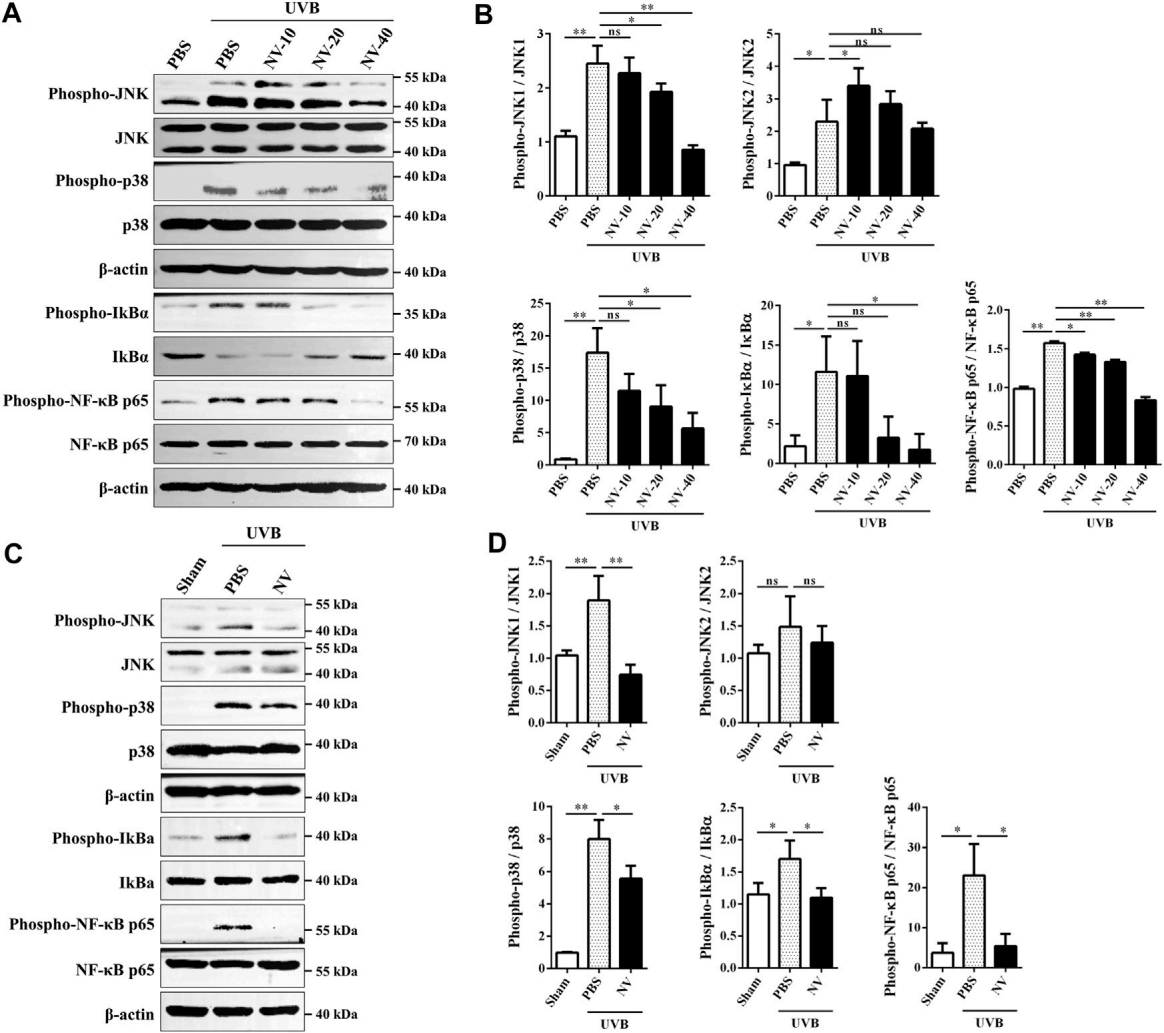
Effects of antioxidin-NV on MAPKs and NF-κB signaling pathways. **(A**,**B)** Western blot showed effects of antioxidin-NV on JNK, p38, IkBα and p65 protein kinases phosopholyation in HaCaT cells **(A)** and relative activation analysis **(B)**. **(C**,**D)** Western blot showed effects of antioxidin-NV on JNK, p38, IkBα and p65 protein kinases phosopholyation in skin tissues **(C)** and relative activation analysis **(D)**. The results were quantified by Image J. The densitometry of phosphorylated JNK, p38, IkBα and p65 were normalized to total JNK, p38, IkBα and p65, and graphed as the mean ± SD (*n* = 3). NV-10, 20, 40 indicated the concentrations of 10, 20, 40 μg/ml respectively. ns, no significance, ^*^ *p* < 0.05, ^**^ *p* < 0.01.

MAPKs and NF-κB signaling are known to be important signal transduction pathways activated by UVB irradiation (Subedi et al., 2017). Therefore, western blot analysis was performed to further explore the effect of antioxidin-NV on MAPK and NF-κB signaling pathway in HaCaT cells and skin tissues. As illustrated in Figures 8A,B, UVB irradiation markedly increased JNK, p38, IkBα and p65 phosphorylation in HaCaT cells, but antioxidin-NV significantly decreased UVB-induced JNK, p38, IkBα and p65 phosphorylation in a concentration-dependent manner. The same results were observed in UVB-irradiated hairless mice, antioxidin-NV also significantly decreased JNK, p38, IkBα and p65 phosphorylation (Figures 8C,D).

### Antioxidin-NV Rescued Collagen Production in UVB-Irradiated Hairless Mice by Rescuing a-SMA Accumulation and Restoring TGF-β1/Smad2 Signaling

**FIGURE 9:**
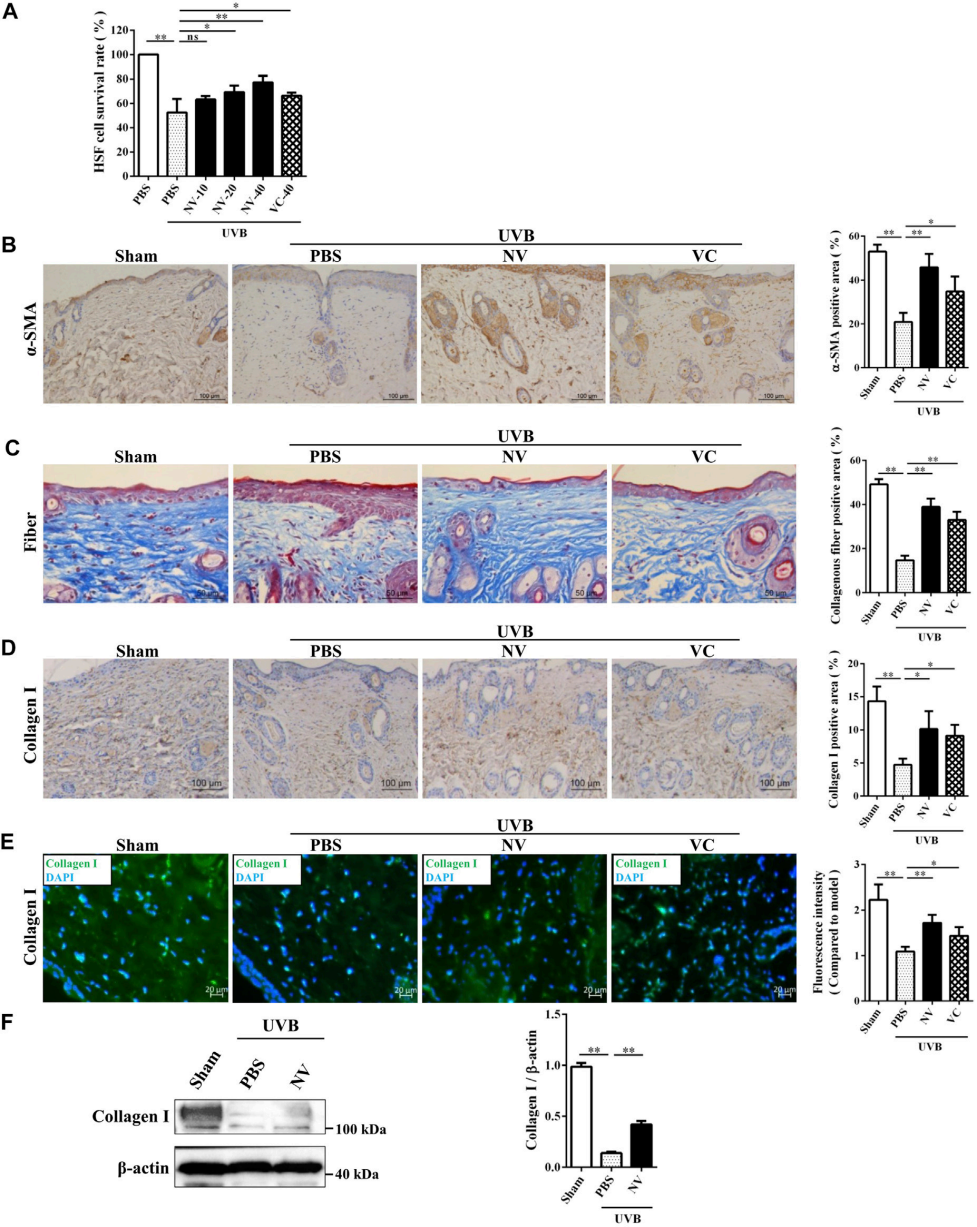
Antioxidin-NV rescued a-SMA accumulation and increased the expression of type I collagen in hairless mice. **(A)** Cultured HSF cells were treated with antioxidin-NV with indicated concentration, and the survival rate of cells were estimated. **(B)** The skin tissues were taken and paraffin blocks were cut into 4 μm thick sections for α-SMA immunohistochemical. Myofibroblast are stained brown (Indicated by red arrow). Scale bar = 100 μm. Quantification of α-SMA-positive area. **(C)** Masson’s trichrome staining estimated for relative collagen density and Quantification of collagen-positive area. Collagenous fiber is stained blue. Scale bar = 50 μm. **(D)** IHC staining of skin tissues was used to determine the expression levels of Collagen I. Collagen I are stained brown (Indicated by red arrow). Scale bar = 100 μm. **(E)** Immunofluorescence staining of skin tissues was used to determine the expression levels of Collagen I and immunofluorescence intensity analysis. Collagen I is stained green. Scale bar = 20 μm. **(F)** Western blot analysis of protein expression in skin tissues and relative expression analysis. Data are presented as mean ± SD (*n* = 3). NV-10, 20, 40 indicated the concentrations of 10, 20, 40 μg/ml respectively. ns, no significance, ^*^ *p* < 0.05, ^**^ *p* < 0.01.

HSFs, which can synthesize and maintain the extracellular matrix of skin and reduce skin photoaging, are a very critical cell type in skin photoaging (Tobin, 2017). Given the above observation that antioxidin-NV significantly suppressed UVB-induced skin photoaging in hairless mice, we further explored the potential effect of antioxidin-NV on HSF cells survival rate and the accumulation of alpha smooth muscle actin (a-SMA) following UVB irradiation *in vitro* and *in vivo*. As illustrated in Figure 9A, UVB irradiation directly inhibited HSF survival rate, but antioxidin-NV obviously restored HSF cells survival rate post UVB irradiation in a concentration-dependent manner. The expression of α-SMA, a marker protein of HSF cells was examined in the skin tissue of UVB-irradiated mice using IHC staining. As illustrated in Figure 9B, UVB irradiation obviously reduced the expression of α-SMA in the skin of UVB-exposed mice, but a higher accumulation of a-SMA positive staining was observed in NV treatment when compared to PBS.

Collagen is derived from HSF cells and plays important role in maintaining the elasticity of skin (Lee et al., 2012). Considering that antioxidin-NV has strong ability to restore the accumulation of a-SMA in hairless mice following UVB irradiation, and a-SMA is also a marker of myofibroblast which has a higher capacity to synthesize collagen (Nakyai et al., 2018). We further investigated whether antioxidin-NV can promote collagen production in UVB-irradiated hairless mice. Masson’s trichrome staining was used to evaluate the presence and distribution of collagen. As shown in Figure 9C, the collagen fibers of mice without UVB irradiation (sham) were dense and regular, while the collagen fibers of mice became less dense and more erratically arranged after UVB irradiation, but antioxidin-NV treatment markedly increased the abundance and density of collagen fibers in UVB-irradiated skins compared to PBS treatment. Type I collagen, which is the major component of collagen fibrils, is the most abundant structural protein in the skin (Makrantonaki and Zouboulis, 2007). Therefore, we further explored the potential effect of antioxidin-NV on type I collagen expression in the skin. Collagen I expression level in the skin tissue was examined using IHC staining (Figure 9D), immunofluorescence (Figure 9E) and western blot (Figure 9F). The results showed that UVB irradiation markedly resulted in a reduction of collagen I deposition in the skin of hairless mice, while antioxidin-NV treatment significantly rescued collagen I production in hairless mice post UVB irradiation (Figures 9D–F).

**FIGURE 10:**
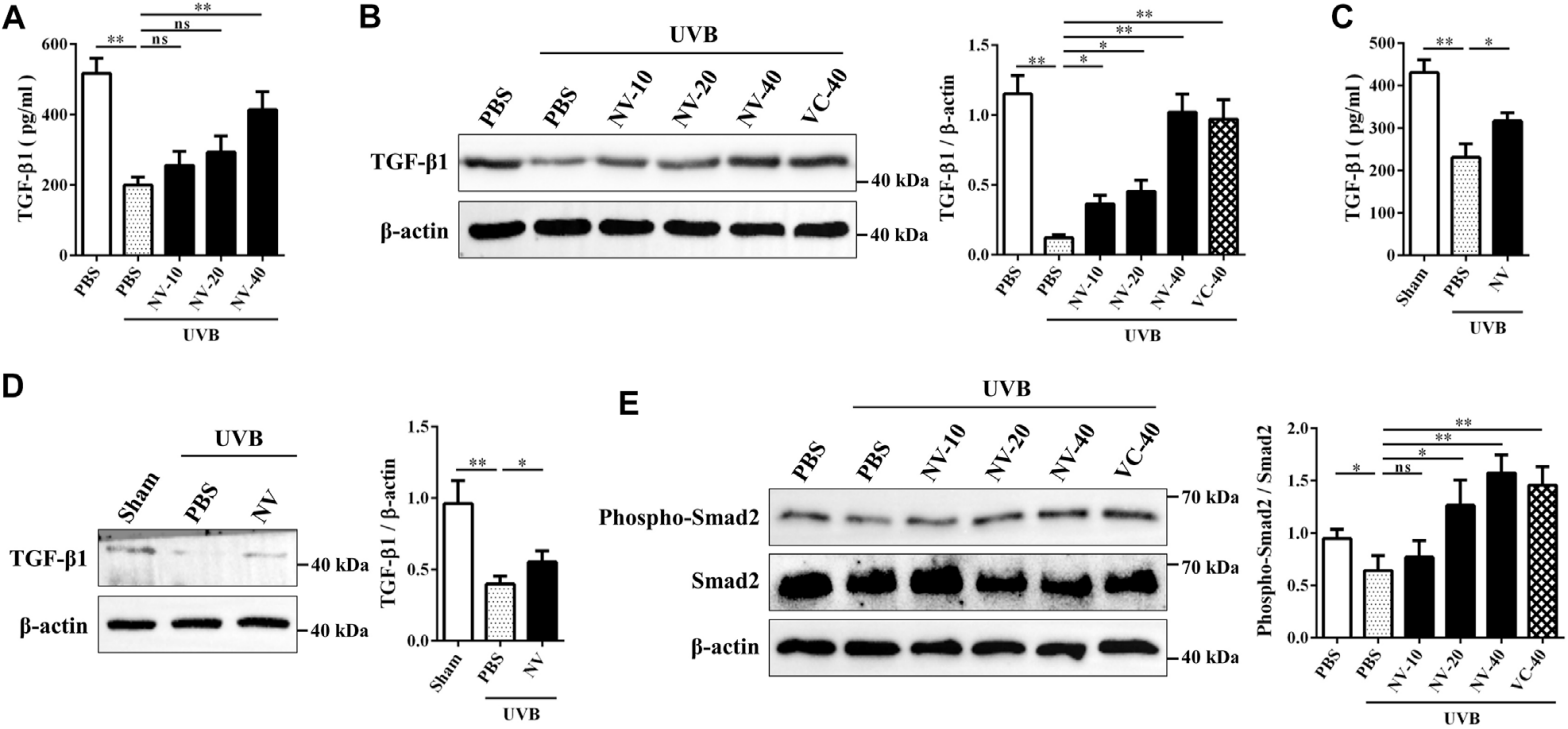
Antioxidin-NV restored TGF-β1 secretion and TGF-β/Smad2 signaling pathway in HSF cells. **(A**,**B):** ELISA **(A)** and Western blot **(B)** showed antioxidin-NV significantly increased TGF-β1 secretion in HSF cells. **(C**,**D)** ELISA **(C)** and Western blot **(D)** showed antioxidin-NV significantly increased TGF-β1 secretion in hairless mice skin. **(E)** Western blot showed effects of antioxidin-NV on TGF-β signaling pathways and relative activation analysis. The results were quantified by Image J. The densitometry of phosphorylated Smad2 were normalized to Smad2, and graphed as the mean ± SD (*n* = 3). NV-10, 20, 40, VC-40 indicated the concentrations of 10, 20, 40 μg/ml respectively. ns, no significance, ^*^ *p* < 0.05, ^**^ *p* < 0.01.

Transforming growth factor-β (TGF-β) is an important cytokine that promotes collagen production (Mouw et al., 2014). In addition, the TGF-β/Smad pathway also promotes the differentiation of myofibroblasts (Guo et al., 2009). To determine whether antioxidin-NV affect TGF-β secretion in HSF cells upon UVB irradiation, we measured the effect of antioxidin-NV on TGF-β1 production in HSF cells using ELISA and western blot, respectively. UVB irradiation obviously suppressed TGF-β1 production in HSF cells, but antioxidin-NV treatment significantly increased TGF-β1 production in UVB-irradiated HSF compared with PBS treatment in a dose-dependent manner (Figures 10A,B). Furthermore, UVB exposure also suppressed TGF-β1 production in the skin of hairless mice, while topical application of antioxidin-NV significantly increased TGF-β1 production in the skin of UVB-irradiated hairless mice compared with PBS treatment (Figures 10C,D). Furthermore, Smad proteins, including Smad2, are essential components of downstream TGF-β signaling. As illustrated in Figure 10E, UVB irradiation markedly reduced Smad2 phosphorylation in HSF cells, but antioxidin-NV obviously increased Smad2 phosphorylation in UVB-irradiated HSF cells compared to PBS-treated cells in a dose-dependent manner, suggesting that antioxidin-NV rescued collagen production in hairless mice upon UVB exposure through restoring TGF-β1/Smad2 signaling.

## Discussion

Skins are a major target of oxidative stress because of ROS that originate from both endogenous and exogenous sources. Ultraviolet radiation is the most important environmental factor in the development of skin aging that is accompanied by a gradual loss of function, physiological integrity and the ability to cope with internal and external stressors (Bocheva et al., 2019). UVB, in particular, induces biological effects that are 1000 times stronger than UVA (Diffey, 2002). Antioxidant supplementations might be an effective therapeutical strategy to restore skin homeostasis (Portugal et al., 2007; Pham-Huy et al., 2008; Godic et al., 2014). Among vertebrates, skins of amphibian display excellent radio-protective abilities and represent a resource for prospective antioxidant peptides. As a step towards understanding amphibian’s radio-protective ability and identifying novel anti-photoaging peptides, we address this issue and have characterized a potential anti-photoaging peptide (antioxidin-NV) from *N. ventripunctata* skin in this work. The structural organization of antioxidin-NV precursor is similar to amphibian antimicrobial peptide precursors, comprising a highly conserved signal peptide and acidic spacer peptide followed by a variable mature peptide. UVB irradiation causes overproduction of reactive oxygen species (ROS) in the skin, which results in oxidative damage of proteins and nucleic acids, leading to DNA damage, inflammation and apoptosis (Portugal et al., 2007; Baek and Lee, 2016). Our results revealed that topical application of antioxidin-NV greatly suppressed UVB-induced skin erythema, thickness and wrinkle formation in hairless mice, suggesting the peptide has strong therapeutic effects against UVB-induced damage. It has been shown that UVB radiation causes DNA damage such as cyclobutane pyrimidine dimers and six to four pyrimidine-pyrimidone photoproducts (Heffernan et al., 2009), and then the damage induces phosphorylation of the Ser-139 residue of the histone variant H2AX, forming γH2AX. γH2AX is a sensitive molecular marker of DNA damage, and accumulates at the site of damage (Maréchal and Zou, 2013). We observed that UVB induced fragmentation of DNA in HaCaT cells and accumulation of γH2AX signals in the cells and *in vivo*, while our results suggest that antioxidin-NV is beneficial in the prevention of UVB-induced DNA damage *in vivo* and *in vitro*. The effects of antioxidin-NV on DNA damage related signaling pathways need to be further investigated to highlight its protective mechanism *in vitro* and *in vivo*.

Mitochondria are considered the most important source of endogenous ROS in the cell (Gniadecki et al., 2000; Zorov et al., 2014). Excessive ROS leads to oxidative stress that is associated with the mitochondrial uncoupling respiration, formation of the mitochondrial permeability transition pore, and mitochondrial dysfunction (Tiwari et al., 2002; Salimi et al., 2019). Mitochondrial dysfunction and oxidative stress are responsible for the induction or activation of the mitochondrial pathway of apoptosis (Maity et al., 2009). Activation of effector caspases is believed to be the final step in the apoptosis pathways. Among the effector capases, caspase 3 plays a critical role in the execution of apoptosis, because it is required for oligonucleosomal DNA fragmentation and promotes the activation of other effector caspases (Rehm et al., 2002). In this study, because antioxidin-NV significantly suppressed intracellular and mitochondria ROS generation, we hypothesized that antioxidin-NV should possess the anti-apoptotic effect. As expected, antioxidin-NV ameliorated UVB-induced apoptosis and inhibited the expression of apoptosis-specific protein, cleaved caspase 3 in HaCaT cells and skin tissues. Therefore, our results showed that antioxidin-NV could prevent the activation of the mitochondrial pathway of apoptosis by scavenging ROS *in vitro* and *in vivo*. This mechanism differs from that of other agents against skin photoaging by modulating the Nrf2-dependent antioxidant responses (Chaiprasongsuk et al., 2019) or oxidative stress (Zhang et al., 2017).

Inflammation enhances the epidermal hyperproliferative response to UVB and increases production of ROS and cytokines, accelerating the aging process (Pillai et al., 2005). IL-6, a cytokine produced by various cells, such as HSF and HaCaT cells, is a signal molecule that mediates the inflammatory response (Wu et al., 2013) and is also known to be associated with ROS caused by UV radiation. In our study, antioxidin-NV significantly decreased UVB-increased IL-6 secretion *in vitro* and *in vivo*. Furthermore, after the treatment by TLR4 inhibitor MTS510, IL-6 down-regulation induced by antioxidin-NV was completely inhibited. These results indicated that antioxidin-NV inhibited UVB-induced IL-6 expression by blocking TLR4-mediated inflammatory responses, then further decreased the epidermal hyperproliferative response to UVB. UVB-induced ROS production activates MAPKs and NF-κB signaling pathways, which further induce the inflammation and apoptosis in cells and cause skin aging (Subedi et al., 2017). In the present study, antioxidin-NV inhibited UVB-induced MAPK and NF-κB signaling pathway. Antioxidin-NV significantly decreased JNK, p38, IkBα and NF-κB p65 phosphorylation. This demonstrated that JNK, p38, IkBa, and NF-κB p65 signaling pathways were involved in antioxidin-NV-mediated downregulation of inflammatory cytokine production upon UVB irradiation, and they may orchestrate in regulating these cytokines expression and inhibiting skin photoaging process. According to these data, we concluded that antioxidin-NV reduced UVB-induced inflammatory response in HaCaT cells and hairless mice by attenuating UVB-activated TLR4/p38/JNK/NF-κB signaling.

Skin photoaging involves a complex interplay of primarily HaCaT, HSF cells and their associated extracellular matrix. HSF cells are very important factors in skin photoaging, because they can synthesize and maintain the extracellular matrix of skin and reduce skin photoaging (Lee et al., 2012). Antioxidin-NV restored the survival rate of HSF cells upon UVB irradiation in a concentration-dependent manner *in vitro.* Additionally, antioxidin-NV significantly restored UVB-reduced α-SMA expression *in vivo*. The TGF-β pathway regulates aspects of cell growth and extracellular matrix synthesis, including collagen synthesis by dermal HSF cells. TGF-β1, a multifunctional cytokine belonged to TGF-β family members, is a key factor in collagen synthesis, which promotes the expression of collagen and type-I procollagen and inhibits the expression of MMP-1 (Kopecki et al., 2007). Our results indicated that antioxidin-NV increased TGF-β1 secretion *in vitro* and *in vivo.* Antioxidin-NV significantly increased UVB-inhibited TGF-β1 secretion in a dose-dependent manner in HaCaT cells. Antioxidin-NV also significantly upregulated TGF-β1 levels in UVB-induced skin tissues *in vivo*. Furthermore, Smad proteins, including Smad2, are key regulators in TGF-β signaling pathways and they are essential components of downstream TGF-β signaling. Antioxidin-NV activated phosphorylation of Smad2 to increase TGF-β1 secretion *in vitro* and *in vivo*. Collagen derived from HSF cells is one of the main building blocks of skin (Lee et al., 2012). Type I collagen, the major component of collagen fibrils, is the most abundant structural protein in the skin (Makrantonaki and Zouboulis, 2007). Antioxidin-NV significantly rescued the collagen and type I collagen production in HSF cells of the skin tissues after UVB irradiation, and type I collagen rescued by antioxidin-NV is critical for maintaining the elasticity of skin upon UVB irradiation. Therefore, antioxidin-NV rescued collagen production in UVB-irradiated hairless mice by restoring TGF-β1/Smad2 signaling.

In a recent work, two small peptides named FW-1 (FWPLI-NH_2_) and FW-2 (FWPMI-NH_2_) were isolated from the skin secretion of *Hyla annectans*. FW-1 and FW-2 directly inhibited UVB-induced tumor necrosis factor-α (TNF-α) and IL-6 secretion. The authors described that FW-1 and FW-2-mediated downregulation of TNF-α and IL-6 secretion through modulating the UV-induced stress signaling pathways such as MAPKs and NF-κB. Besides, the authors described that FW-1 and -2 displayed antioxidant effects in the skins of mice by reducing UVB-induced ROS production through an unknown mechanism (Liu et al., 2021). In our work, we found that antioxidin-NV directly scavenged free radicals such as ROS and ABTS^+^. Both *H. annectans* in the recent work and *N. ventripunctata* in our work live in the southwestern plateau area of China. This plateau area possesses long duration of sunshine, and suffers strong ultraviolet radiation, which make the naked skin of frogs evolve an effective antioxidant system to scavenge free radicals induced by light radiation. Accordingly, series of peptides have been identified with antioxidant activity from the skin of frogs lived in this plateau area (Yang et al., 2009; Liu et al., 2010), but the previous studies did not investigate whether these peptides have anti-photoaging activity. While our work definitely indicated that antioxidin-NV-mediated reduction of free radical accumulation led to the reduction of DNA damage, apoptosis, and inflammation upon UVB radiation, thereby providing protection against UVB-induced skin pohto-aging. Our study supplement the radio-protective mechanism of frogs lived in the southwestern plateau area of China, and prove the feasibility to identify effective anti-photoaging peptide from the frogs lived this plateau area.

In conclusion, antioxidin-NV identified from *N. ventripunctata* skin is a bioactive/effector compound with potential anti-photoaging ability. It shows strong antioxidant activities by scaveging intracellular and mitochondrial ROS accumulation upon ultraviolet radiation. As subsequent results, it inhibits UVB-induced DNA damage, apoptosis, and inflammation. Our results suggest that the therapeutic effect of antioxidant-NV on UV-induced photoaging was mediated through the alleviation of the oxidative stress-induced process of skin photoaging. Thus, antioxidant-NV may serve as a potent candidate for the prevention and therapy of photoaging.

## Data Availability

The datasets presented in this study can be found in online repositories. The names of the repository/repositories and accession number(s) can be found in the article/Supplementary Material.

## Abbreviations

ABTS^+^, 2,2-Azinobis (3-Ethylbenzothiazoline-6-Sulfonic Acid); DMEM, dulbecco’s modified eagle medium; ELISA, enzyme-linked immunosorbent assay; ERK, extracellular regulated protein kinases; FBS, fetal bovine serum; HaCaTs, Human Immortalized Keratinocytes; HSFs, human skin fibroblasts; IκBα, Nuclear Factor kappa-B Inhibitor alpha; IL-6, Interleukin-6; JNK, c-Jun NH2-terminal kinase; MALDI-TOF, matrix-assisted laser desorption ionization time-of-flight; MAPK, mitogen-activated protein kinases; MTT, 3-(4, 5-dimethylthiazol-2-yl)-2,5-diphenyl-2H-tetrazolium bromide; NF-κB, Nuclear Factor kappa-B; NV, antioxidin-NV; *N. ventripunctata*, *Nanorana ventripunctata*; p38, p38 mitogen-activated protein kinases; PBS, phosphate-buffered saline; ROS, Reactive Oxygen Species; RP-HPLC, reversed-phase high performance liquid chromatography; α-SMA, α smooth muscle actin; sNV, scrambled antioxidin-NV; TGF-β1, transforming growth factor beta1; UVB, Ultraviolet Radiation B; VC, vitamin C.
