# Supplementary material for: A Frog Peptide Ameliorates Skin Photoaging Through Scavenging Reactive Oxygen Species
Source: Front Pharmacol. 2022 Jan 19;12:761011. doi: 10.3389/fphar.2021.761011 (PMC8807480; doi:10.3389/fphar.2021.761011)
Supplement: Supplementary file 1 [file DataSheet1.DOCX]

**Supplementary Material**

**
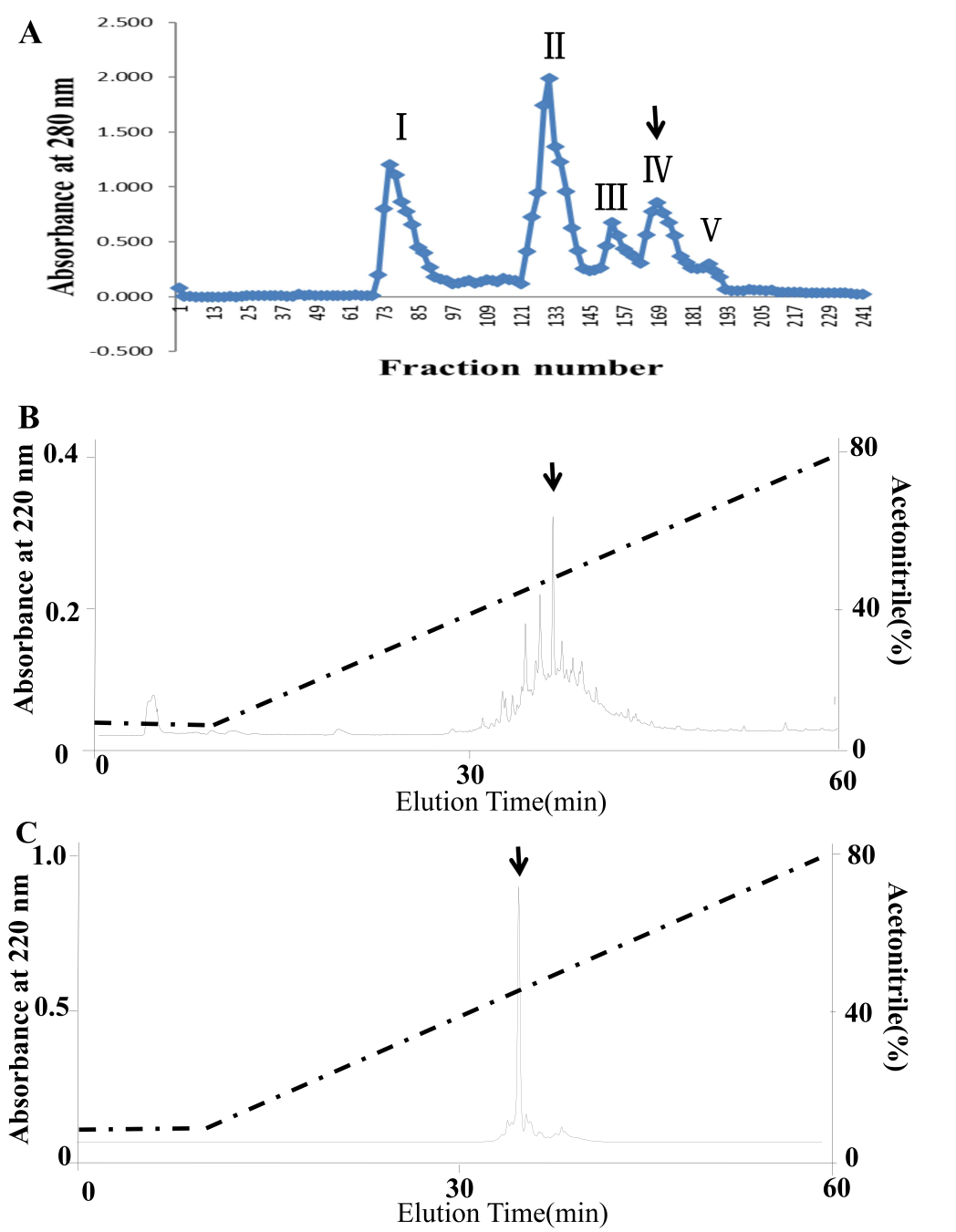
**

**FIGURE S1. Purification of antioxidin-NV** **from the skin secretions of *N. ventripunctata.* A:** Sephadex G-50 gel filtration of skin secretions of N. ventripunctata. The skin secretions were applied to Sephadex G-50 gel filtration column. The elution was performed by 0.1M PBS, collecting raction of 3.0 ml. The fraction containing cell proliferation activity is marked by an arrow. **B:** The interesting fraction from the Sephadex G-50 gel filtration was further purified by C_18_ RP-HPLC column. The elution was performed at a flow rate of 0.7 ml/min with the indicated gradient of acetonitrile in 0.1% (v/v) trifluoroacetic acid (TFA) in water. **C:** The eluted peak (*arrow* in **B**) containing antioxidative activity was further purified by C_18_ RP-HPLC column. The purified antioxidin-NV is indicated by an *arrow.*


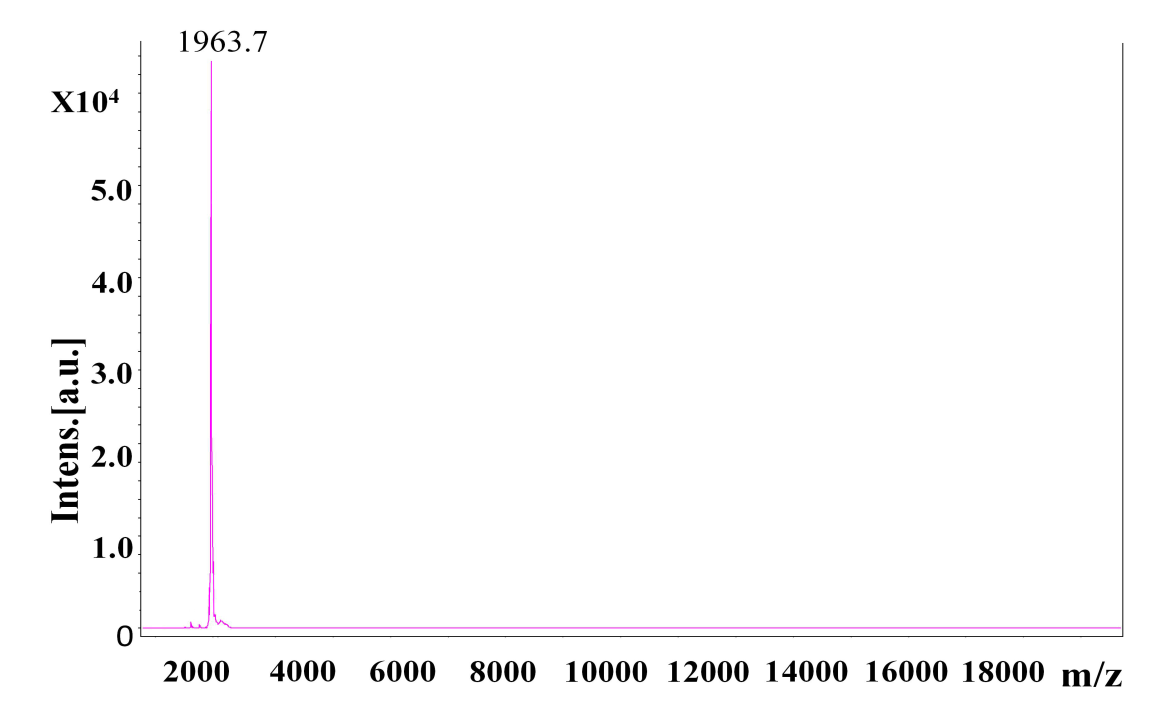


**FIGURE S2. MALDI-TOF mass spectrometry analysis of antioxidin-NV.**


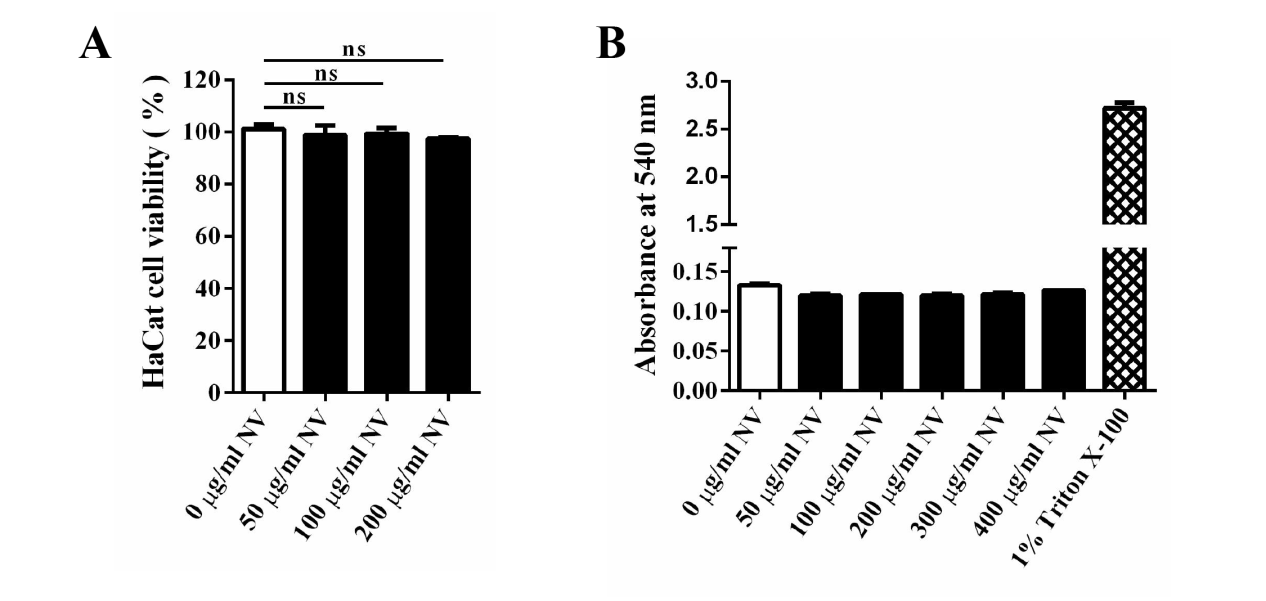


**FIGURE S3. Antioxidin-NV exhibited no cytotoxicity and hemolytic activity. A:** The effect of Antioxidin-NV on HaCat cytotoxicity. **B:** Verification of the hemolytic activity of Antioxidin-NV on rabbit blood. The antioxidin-NV at concentrations of 0, 50, 100, 200, 300, 400 μg/ml was incubated with rabbit red blood cells at 37°C for 30 min. Normal saline was used as the negative treatment group, 1% Triton X-100 was used as the positive control, and the absorbance at 540 nm was measured. Data are presented as mean ± SD (n = 5). ns, no significance.

**Table S1. Primer sequences used for cloning in this study**

| **Primer** | **Sequence (5’ 3’)** | **application** |
| --- | --- | --- |
| Antioxidin-NV-R_1_ | TTIARIGTRTTNGCCCANCC* | 5’ end screening |
| 5’ PCR primer | AAGCAGTGGTATCAACGCAGAGT | 5’ end screening |
| Antioxidin-NV-F_1_ | ATGTTCACCTTGAAGAAGTCCCT | 3’ end screening |
| 3’ PCR primer | CGGGGTACGATGACACCA | 3’ end screening |
| *Where Y stands for C or T; R stands for A or G; N stands for A,C,G or T. | | |
